# Receptor-interacting protein 1 and 3 kinase activity are required for high-fat diet induced liver injury in mice

**DOI:** 10.3389/fendo.2023.1267996

**Published:** 2023-12-15

**Authors:** Xiaoqin Wu, Rakesh K. Arya, Emily Huang, Megan R. McMullen, Laura E. Nagy

**Affiliations:** ^1^ Northern Ohio Alcohol Center, Department of Inflammation and Immunity, Cleveland Clinic, Cleveland, OH, United States; ^2^ Department of Gastroenterology and Hepatology, Cleveland Clinic, Cleveland, OH, United States; ^3^ Department of Molecular Medicine, Case Western Reserve University, Cleveland, OH, United States

**Keywords:** RIP1 kinase, RIP3 kinase, cell death, NAFLD, obesity, FFC diet

## Abstract

**Background:**

The RIP1-RIP3-MLKL-mediated cell death pathway is associated with progression of non-alcohol-associated fatty liver/steatohepatitis (NAFL/NASH). Previous work identified a critical role for MLKL, the key effector regulating necroptosis, but not RIP3, in mediating high fat diet-induced liver injury in mice. RIP1 and RIP3 have active N-terminus kinase domains essential for activation of MLKL and subsequent necroptosis. However, little is known regarding domain-specific roles of RIP1/RIP3 kinase in liver diseases. Here, we hypothesized that RIP1/RIP3 kinase activity are required for the development of high fat diet-induced liver injury.

**Methods:**

*Rip1^K45A/K45A^* and *Rip3^K51A/K51A^* kinase-dead mice on a C57BL/6J background and their littermate controls (WT) were allowed free access to a diet high in fat, fructose and cholesterol (FFC diet) or chow diet.

**Results:**

Both *Rip1^K45A/K45A^* and *Rip3^K51A/K51A^* mice were protected against FFC diet-induced steatosis, hepatocyte injury and expression of hepatic inflammatory cytokines and chemokines. FFC diet increased phosphorylation and oligomerization of MLKL and hepatocyte death in livers of WT, but not in *Rip3^K51A/K51A^*, mice. Consistent with *in vivo* data, RIP3 kinase deficiency in primary hepatocytes prevented palmitic acid-induced translocation of MLKL to the cell surface and cytotoxicity. Additionally, loss of *Rip1* or *Rip3* kinase suppressed FFC diet-mediated formation of crown-like structures (indicators of dead adipocytes) and expression of mRNA for inflammatory response genes in epididymal adipose tissue. Moreover, FFC diet increased expression of multiple adipokines, including leptin and plasminogen activator inhibitor 1, in WT mice, which was abrogated by *Rip3* kinase deficiency.

**Discussion:**

The current data indicate that both RIP1 and RIP3 kinase activity contribute to FFC diet-induced liver injury. This effect of RIP1 and RIP3 kinase deficiency on injury is consistent with the protection of *Mlkl^-/-^* mice from high fat diet-induced liver injury, but not the reported lack of protection in *Rip3^-/-^* mice. Taken together with previous reports, our data suggest that other domains of RIP3 likely counteract the effect of RIP3 kinase in response to high fat diets.

## Introduction

Upon activation of death receptor signaling, receptor-interacting protein kinases 1 (RIP1) and 3 (RIP3) play critical roles in the classical pathway of necroptotic cell death, leading to phosphorylation of mixed lineage kinase domain-like (MLKL), the effector molecule in necroptosis. Canonical RIP1-RIP3-MLKL-mediated necroptosis is implicated in multiple human diseases, including in the progression of non-alcohol-associated fatty liver/steatohepatitis (NAFL/NASH) (1–3). Studies using RIP1 kinase inhibitors and RIP1 kinase dead mice have consistently identified the important role of RIP1 kinase activity in multiple dietary models of fatty liver disease. For example, RIPA-56, a highly specific inhibitor of RIPK1, reduced high fat diet (HFD)-induced liver steatosis and injury partly through an increase in mitochondrial respiration (4). Another recent study found that genetic inhibition of RIP1 kinase reduced hepatic cell death and inflammation and alleviated hepatic steatosis, liver damage and fibrosis in both HFD and methionine-choline deficient (MCD) dietary models (5).

In contrast, RIP3, the direct target of RIP1 kinase, plays differential roles in multiple dietary models of fatty liver disease. RIP3 expression is not detectable in healthy hepatocytes (6–8), associated with epigenetic suppression (6). However, expression is detected in hepatocytes in patients with alcohol-associated liver disease (9) and in murine hepatocytes in response to chronic ethanol feeding (9, 10) and FFC-diet induced liver injury (8), but not in response to choline deficient high fat or 60% high fat diets (6). *Rip3^-/-^* mice are protected from ethanol (9, 10) and MCD diet-induced liver injury (11); while *Rip3* deficiency did not protect mice in two different models of high-fat diet induced liver injury: 1) the traditional high-fat diet (HFD) model (12) and 2) the Western diet model utilizing a diet high in fat, fructose and cholesterol (FFC diet) (8, 13). Interestingly, *Rip3* knockout mice on a combined choline deficient (CD)-HFD diet developed more pronounced glucose intolerance and adipose tissue inflammation and liver injury than WT littermates (14). Taken together, previous work indicates that the roles of RIP1 and RIP3 in murine models of NAFL/NASH are complex and not completely understood.

RIP1 and RIP3 have an active N-terminus kinase domain essential for activation of MLKL in the classical necroptotic pathway. In addition to regulating cell death, RIPK1 and RIPK3 kinases are implicated in the regulation of inflammatory responses. For example, both RIP1 and RIP3 kinases promote sustained activation of ERK, cFos and NFκB required for acute inflammatory response (15). Moreover, RIP3 kinase activity is essential for TLR-induced NLRP3 activation in the absence of both IAPs and caspase-8 (16). We have previously reported that FFC diets increase the phosphorylation, oligomerization and translocation of MLKL to the plasma membrane. *Mlkl*-deficient are protected from FFC diet-induced tissue injury, but, surprisingly, *Rip3-*deficient mice are not protected (8, 13). These studies suggest that MLKL can be activated by RIP3- independent mechanisms and/or that there are domain-specific roles of RIP1/RIP3 kinase in liver diseases.

Here we hypothesized that the kinase domain in RIP1 and RIP3 are required for development of FFC diet-induced liver injury. *Rip1* (*Rip1* KDKI, *Rip1^K45A/K45A^*) and *Rip3* (*Rip3* KDKI, *Rip3K51A/K51A* ^)^ kinase dead knock-in mice and littermate (wild type, WT) controls were fed chow or FFC diet for 12 weeks. FFC diet increased the phosphorylation and oligomerization of MLKL in livers of WT, but not in *Rip3^K51A/K51A^*, mice. Importantly, *Rip1^K45A/K45A^* and *Rip3^K51A/K51A^* mice were protected against FFC diet-induced liver injury and inflammatory responses in adipose. Together, these data indicate that RIP1 and RIP3 kinase activity contributes to FFC diet-induced injury. This is consistent with the protection of *Mlkl^-/-^* mice from Western diet-induced liver injury, but not the reported lack of protection in *Rip3^-/-^* mice. Taken together with previous reports, our data suggest that other domains of RIP3 likely counter the role of RIP3 kinase in response to FFC diet.

## Materials and methods

### Animals and FFC diet feeding

All procedures using animals were approved by the Cleveland Clinic Institutional Animal Care and Use Committee. *Rip1^K45A/K45A^* and *Rip3^K51A/K51A^* mice were originally generated by GlaxoSmithKline. Briefly, *Rip1^K45A/K45A^* and *Rip3^K51A/K51A^* mice were generated through homologous recombination, utilizing targeting construct that mutated the catalytic lysine residue to alanine (K45A and K51A, respectively), resulting in the complete elimination of kinase activity (17, 18). Both strains are viable and fertile backcrossed to a C57BL/6J background and WT controls were wild-type littermates.

Male mice (5-6 weeks of age) were allowed free access to a chow diet (Chow) containing 6% fat/13.0 kJ/g (#2918, Teklad Mills, Madison, WI) or FFC diet (AIN-76A Western diet, #5342, TestDiet, St. Louis, MO) for 12 weeks. The FFC diet contained 40% energy as fat (12% saturated fatty acid, 0.2% cholesterol) with fructose and glucose added to the water (42 g/L final concentration).

Food intake was similar between genotypes in response to FFC diet. As expected, body and liver weights were higher with FFC feeding in all genotypes compared to chow-fed mice. *Rip1^K45A/K45A^* and *Rip3^K51A/K51A^* mice gained 12% and 11% less, respectively, on the FFC diet compared to WT (**Supplementary Figure S1**).

### Subcellular fractionation and plasma membrane isolation from murine liver

Plasma fractions were isolated using the plasma membrane protein extraction kit (ab65400, Abcam). Liver tissues were resuspended in homogenization buffer and lysed using a Dounce homogenizer (50-60 strokes). Homogenates were centrifuged to obtain the cytosolic fraction as well as the 10,000g pellet (total membrane fraction). The pellet was further purified according to the manufacturer’s instructions to obtain plasma membrane enriched proteins. Additional Materials and Methods are provided the **Supplementary Material**.

### Statistical analysis

Values shown in all figures represent the means ± SEM. Analysis of variance was performed using the general linear model’s procedure (SAS, Carey, IN). Data were log-transformed as necessary to obtain a normal distribution. Follow-up comparisons were made by least square means testing. *P* values of less than 0.05 were considered significant. Values with different superscripts are significantly different from each other, *P <*0.05.

## Results

### Both RIP1 and RIP3 kinase deficiency protect mice from FFC diet-induced liver steatosis, inflammation and injury

**Figure 1:**
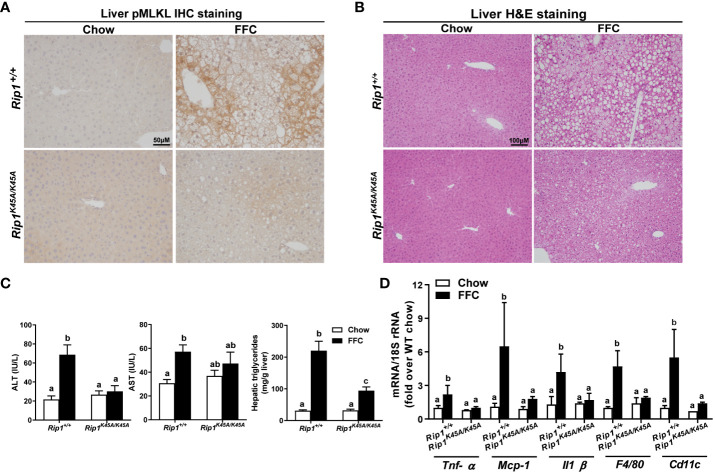
RIP1 kinase activity was required for FFC-induced phosphorylation of MLKL and liver injury. *Rip1^+/+^* and *Rip1^K45A/K45A^* mice were allowed free access to chow or FFC diet for 12 weeks. **(A)** Immunohistochemistry staining for pMLKL in paraffin-embedded liver sections. Images were acquired using a 10X objective. Representative images are shown from n=5-6 per group. **(B)** Hematoxylin and eosin (H&E) staining of liver sections. Images were acquired at 10X magnification. **(C)** ALT/AST concentration in plasma and hepatic triglyceride content in liver homogenates. **(D)** mRNA expression of genes of interest in livers was detected by qRT-PCR. Values with different alphabetical superscripts are significantly different from each other, n = 5-6 per group. p <0.05, assessed by ANOVA.

RIP1, upstream of RIP3 in the pathway of necroptosis, is a multitasking molecule harboring distinct roles with kinase-dependent/independent functions (19, 20). In order to study whether RIP1 kinase activity is required in a dietary model of liver injury, *Rip1^K45A/K45A^* mice and WT were allowed free access to chow or FFC diet for 12 weeks. FFC diet feeding increased phosphorylation of MLKL in liver of WT, but not *Rip1^K45A/K45A^*, mice (**Figure 1A**). Consistent with a previous study in a different dietary model of NAFL/NASH (5), *Rip1^K45A/K45A^* mice were protected from FFC diet-induced liver injury. Absence of *Rip1* kinase prevented FFC diet-mediated hepatic steatosis (**Figure 1B**), increased activity of ALT/AST in the circulation (**Figure 1C**), hepatic triglyceride accumulation (**Figure 1C**) and pro-inflammatory responses (**Figure 1D**).

**Figure 2:**
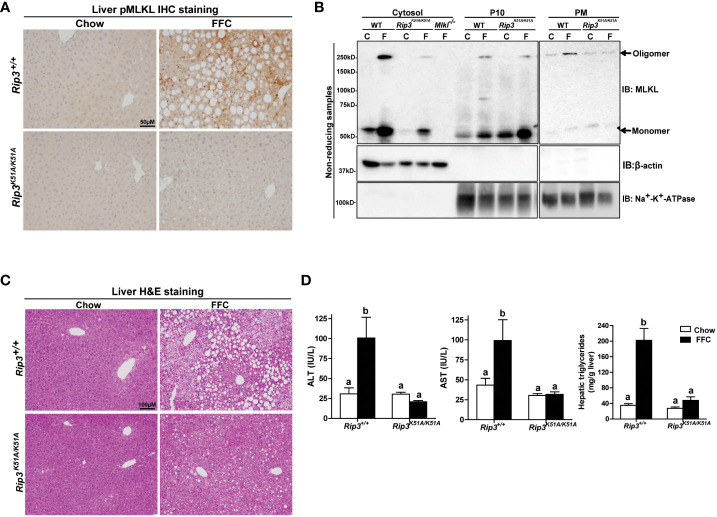
RIP3 kinase activity was required for FFC-induced phosphorylation and oligomerization of MLKL in mouse liver and liver injury. *Rip3^+/+^* and *Rip3^K51A/K51A^* mice were allowed free access to chow or FFC diet for 12 weeks. **(A)** Immunohistochemistry staining for pMLKL in paraffin-embedded liver sections. Images were acquired using a 10X objective. Representative images are shown from n=5-6 per group. **(B)** Subcellular fractions (cytosol, P10 (10,000 g pellet) and plasma membrane (PM) were isolated from liver, proteins separated by SDS-PAGE under non-reducing condition, and immunoblotted (IB) with anti-MLKL antibody. Enrichment of sub-cellular fractions was verified by probing for Na^+^/K^+^-ATPase (plasma and intracellular membrane marker) and β-actin (cytosolic marker). Blots are representative from n = 3 for PM and 6 for cytosol and P10 independent experiments. Western blots were semi-quantified on Image J. Values for MLKL oligomer in the P10 fraction were 0.05 ± 0.02^a^ for chow-fed and 0.32 ± 0.14^b^ for FFC-fed in *Rip3^+/+^* and 0.05 ± 0.03^a^ for chow-fed and 0.12 ± 0.04^a^ for FFC-fed in *Rip3^K51A/K51A^* (p<0.05). Values for MLKL oligomer in the PM fraction were 0.07 ± 0.03^a^ for chow-fed and 0.24 ± 0.06^b^ for FFC-fed in *Rip3^+/+^* and 0.05 ± 0.02^a^ for chow-fed and 0.07 ± 0.02^a^ for FFC-fed in *Rip3^K51A/K51A^* (p<0.05). **(C)** Hematoxylin and eosin (H&E) staining of liver sections. Images were acquired at 10X magnification. **(D)** ALT/AST concentration in plasma and hepatic triglyceride content in liver homogenates. Values represent means ± SEM. Values with different alphabetical superscripts are significantly different from each other, n = 5-6 per group. p <0.05, assessed by ANOVA.

**Figure 3:**
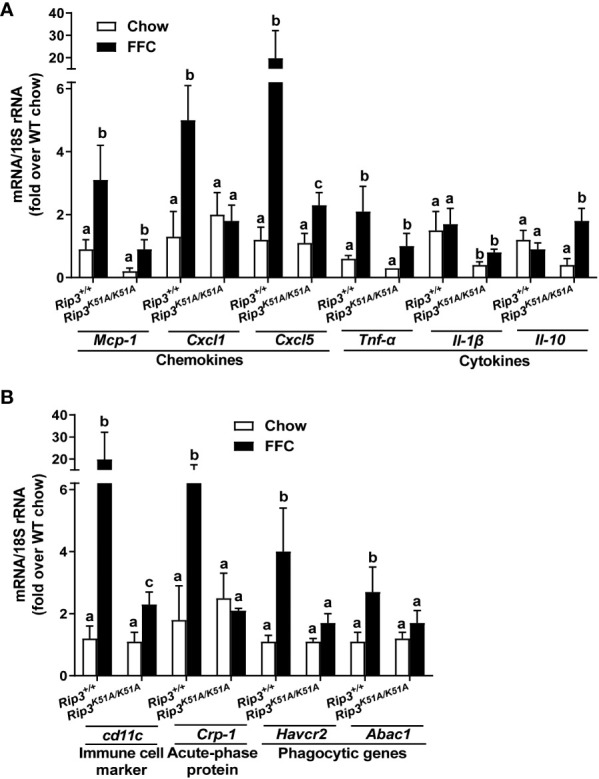
RIP3 kinase activity contributed to FFC-induced liver inflammatory responses. *Rip3^+/+^* and *Rip3^K51A/K51A^* mice were allowed free access to chow or FFC diet for 12 weeks. **(A)** Expression of chemokine (*Mcp-1, Cxcl1* and *Cxcl5*) and cytokine (*Tnf-α*, *Il-1β* and *Il-10*) mRNA in livers. **(B)** Expression of immune cell marker *Cd11c*, acute-phase protein *Crp-1, and phagocytic gene (Havcr2*, *Abca1)* mRNA in livers. Expression was normalized to 18S rRNA and expressed as the percent increase over chow-fed controls. Values represent means ± SEM. Values with different superscripts are significantly different from each other, n = 5-6 per group. p <0.05, assessed by ANOVA.

Immunohistochemical analysis revealed that FFC diet feeding also increased phosphorylation of MLKL in liver of WT, but not *Rip3K51A/K51A*, mice (**Figure 2A**). Since RIP3 is the proximal kinase in the phosphorylation of MLKL, we also investigated the subcellular localization of MLKL in WT and *Rip3K51A/K51A* mice in response to FFC diet. FFC diet induced the translocation and oligomerization of MLKL at the plasma membrane in WT, but not *Rip3* kinase deficient, mice (**Figure 2B**), indicating that the functional activation of MLKL in response to FFC diet requires RIP3 kinase activity. *Rip3K51A/K51A* mice were also protected from FFC diet-induced liver injury. Absence of *Rip3* kinase prevented FFC diet-mediated hepatic steatosis (**Figure 2C**), increased activity of ALT/AST in the circulation (**Figure 2D**) and accumulation of hepatic triglycerides (**Figure 2D**). Expression of mRNA for a number of pro-inflammatory chemokines and cytokines (**Figure 3A**) and immune cell markers, the acute phase protein *Crp-1* and phagocytic-related genes (**Figure 3B**) were also increase in WT, but not *Rip3* kinase deficient, mice. In contrast, expression of mRNA for the anti-inflammatory cytokine *Il-10* was higher in *Rip3^K51A/K51A^* mice compared to WT mice in response to FFC diet (**Figure 3A**).

**Figure 4:**
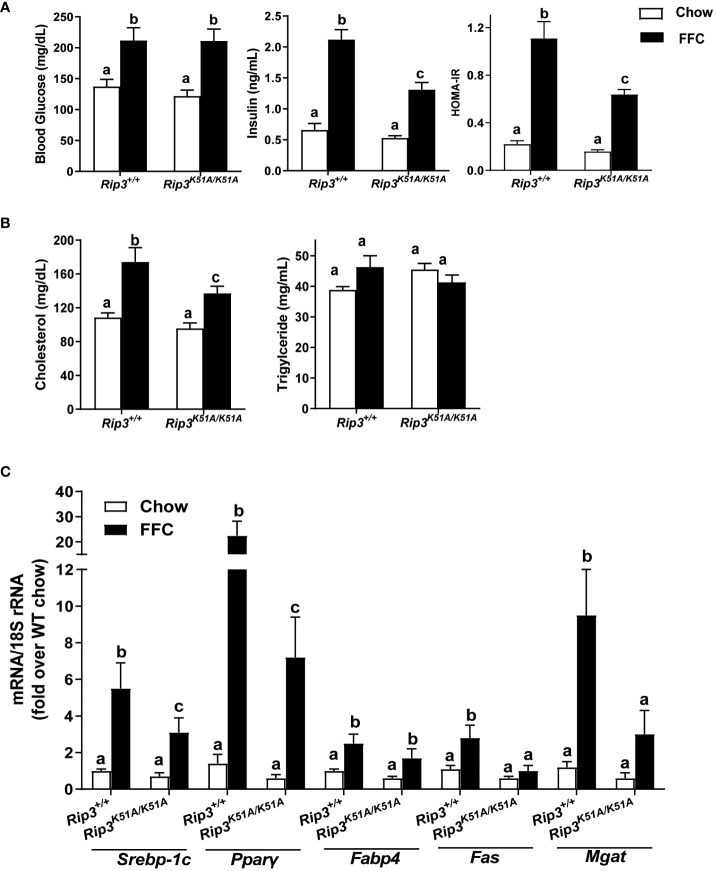
RIP3 kinase activity was involved in FFC-induced metabolic disturbances and regulation of genes involved in lipogenesis. *Rip3^+/+^* and *Rip3^K51A/K51A^* mice were allowed free access to chow or FFC diet for 12 weeks. **(A)** Plasma concentrations of blood glucose, insulin and calculated HOMA-IR, as well as **(B)** cholesterol and triglycerides in plasma from *Rip3^+/+^* and *Rip3^K51A/K51A^* mice. **(C)** Expression of lipogenesis-related mRNA including *Srebp-1c*, *Pparγ*, *Fabp4, Fas* and *Mgat*, in the liver was determined by qRT-PCR. Expression was normalized to 18S rRNA and expressed as the percent increase over chow-fed controls. Values represent means ± SEM. Values with different superscripts are significantly different from each other, n = 5-6 per group. p <0.05, assessed by ANOVA.

Insulin resistance is a common feature of NAFL/NASH (21). FFC diet increased fasting glucose concentrations in WT and *Rip3K51A/K51A* mice compared to chow-fed mice independently of genotype (**Figure 4A**). However, fasting insulin concentrations and calculated Homeostatic Model Assessment for Insulin Resistance (HOMA-IR) were increased with FFC feeding in WT, but not *Rip3^K51A/K51A^*, mice (**Figure 4A**). Plasma indicators of lipid homeostasis were also differentially impacted by genotype. FFC diet increased plasma cholesterol and TG in WT mice; this increase in plasma cholesterol, but not TG, was reduced in *Rip3^K51A/K51A^* mice (**Figure 4B**). *Rip3* kinase deficiency also prevented FFC diet-induced accumulation of multiple lipogenesis-related genes, including *Fabp4*, *Fas*, *Mgat*, *Pparγ* and *Srebp-1c* (**Figure 4C**). Together, these data indicate that the kinase activity of RIP3 contributes to FFC diet-induced metabolic perturbations and liver injury.

### *Rip3* kinase deficiency protects hepatocytes from FFC-induced hepatocyte death and palmitic acid-mediated cytotoxicity

**Figure 5:**
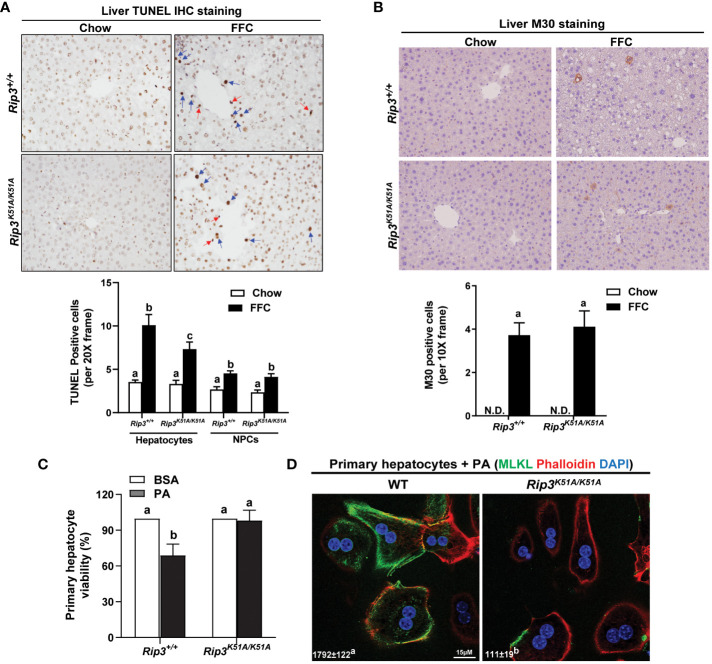
Effect of RIP3 kinase activity in FFC-induced cell death in mouse liver and PA-mediated cytotoxicity in primary hepatocytes. *Rip3^+/+^* and *Rip3^K51A/K51A^* mice were allowed free access to chow or FFC diet for 12 weeks. **(A, B)** Paraffin-embedded livers were de-paraffinized followed by quantitative analyses of **(A)** TUNEL or **(B)** M30 staining. Images were acquired using 20X or 10X objective. The blue arrows indicate TUNEL-positive hepatocytes and red arrows indicate TUNEL-positive NPCs. TUNEL-positive hepatocytes and NPCs were counted and expressed as the total number of cells per 20X frame and M30 positive cells were quantified per 10X frame, n = 5-6 per group. **(C, D)** Primary hepatocytes were isolated from chow-fed WT and *Rip3^K51A/K51A^* mice and then exposed to 500 µM palmitic acid (PA) for 24h. **(C)** MTS assay was performed to determine PA-induced hepatotoxicity and **(D)** location of MLKL at the cell surface (phalloidin: F-actin) in response to PA visualized by confocal microscopy. n=4 independent isolations. Values represent means ± SEM. Values with different superscripts are significantly different from each other, n = 3 per group. *p <*0.05, assessed by ANOVA. N.D., not detectable. TUNEL, Terminal deoxynucleotidyl transferase dUTP nick end labeling.

Hepatocellular death was assessed by both TUNEL and M30 staining in order to distinguish apoptotic and necroptotic cell death; TUNEL staining recognizes both necroptotic/necrotic and apoptotic cells (22) while M30 staining, which detects caspase-cleaved cytokeratin 18, is an indicator of hepatocyte apoptosis (23). Interestingly, while there was no difference in M30^+^ cells between genotypes in response to FFC diet, the number of TUNEL^+^ hepatocytes was higher in WT compared to *Rip3* kinase deficient mice after FFC feeding (**Figures 5A, B**); in contrast, the number of TUNEL^+^ non-parenchymal cells (NPCs) was similar between WT and *Rip3^K51A/K51A^* mice after FFC feeding (**Figure 5A**).

To further assess the contribution of RIP3 kinase activity to hepatocyte death, *in vivo* lipotoxicity was modeled by exposing hepatocytes to PA *in vitro*. PA-induced hepatocyte death is mediated in both caspase- dependent and independent mechanisms (8, 12). Here, *Rip3* kinase deficiency prevented PA-mediated hepatoxicity (**Figure 5C**). Consistent with *in vivo* data, challenge of hepatocytes with PA increased the co-localization of MLKL with the cell surface marker F-actin (phalloidin) in hepatocytes from WT, but not *Rip3^K51A/K51A^*, mice (**Figure 5D**). These data suggest that the kinase activity of RIP3 is involved in regulating FFC diet-induced hepatocyte death and PA-mediated cytotoxicity and MLKL translocation to the membrane.

### Loss of RIP1 or RIP3 kinase activity reduced FFC diet-mediated adipose inflammation

**Figure 6:**
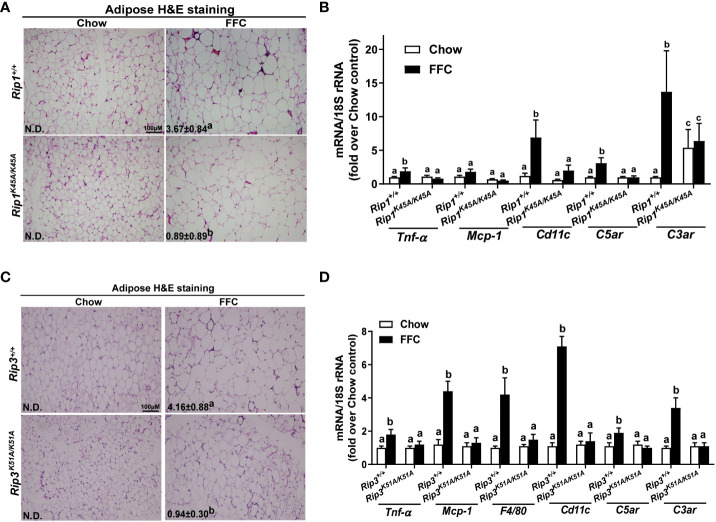
Absence of *Rip1 or Rip3* kinase activity prevented FFC diet-induced inflammatory responses in the adipose. Mice were allowed free access to chow or FFC diet for 12 weeks and epididymal white adipose tissue collected and analyzed. **(A/C)** H&E staining of epididymal white adipose tissues and quantification of crown-like structures per 10X frame in **(A)** *Rip1^+/+^* and *Rip1^K45A/K45A^* mice and **(C)** *Rip3^+/+^* and *Rip3^K51A/K51A^* mice. **(B/D)** Expression of genes of interest were measured by qRT-PCR in adipose tissue from **(B)** *Rip1^+/+^* and *Rip1^K45A/K45A^* mice and **(D)** *Rip3^+/+^* and *Rip3^K51A/K51A^* mice. Values represent means ± SEM. Values with different superscripts are significantly different from each other, n = 5-6 per group. p <0.05, assessed by ANOVA.

Liver-adipose crosstalk plays a critical role in the pathogenesis of NAFLD (24). Adipose tissue dysfunction is associated with adipose inflammation and perturbed secretome (25). Therefore, we investigated whether RIP1 and/or RIP3 kinase impacted FFC diet-mediated adipose inflammation. Crown-like structures, clusters of dead or dying adipocytes surrounded by infiltrating immune cells, are a hallmark of obesity and metabolic syndrome (26). FFC diet increased adipocyte size and the numbers of crown like structures in WT, but not in *Rip1^K45A/K45A^* or *Rip3^K51A/K51A^*, mice (**Figure 6A/C**). Consistent with the morphologic data, FFC diet increased expression of mRNA for cytokines (*Tnf-α* and *Mcp-1*), immune cell markers (*F4/80* and *Cd11c*) and complement receptor *C3ar* to a greater degree in WT compared to *Rip1^K45A/K45A^* or *Rip3^K51A/K51A^* mice (**Figure 6B/D**).

**Figure 7:**
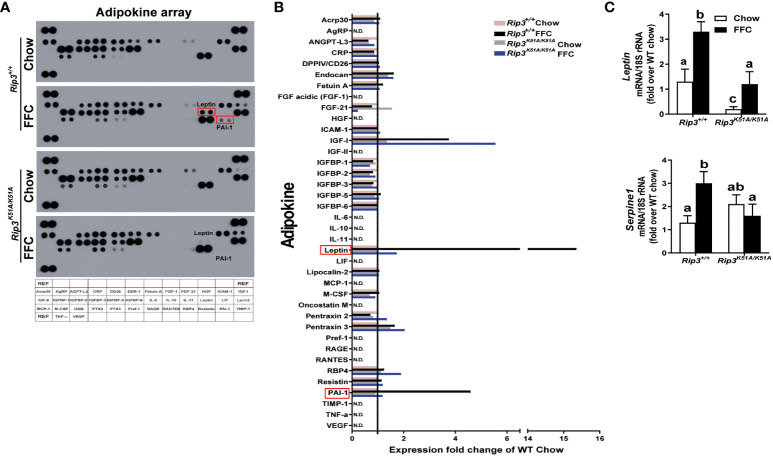
Plasma adipokine profiles in epididymal adipose from *Rip3* kinase deficient mice. **(A)** Mice were allowed free access to chow or FFC diet for 12 weeks and epididymal white adipose tissue collected and analyzed. **(A)** Plasma adipokine profiles were assess using an adipokine array. **(B)** Relative density was calculated using Image J; data were normalized to reference spots, and each experimental group was expressed as fold change of chow-fed *Rip3^+/+^* group. **(C)** Expression of mRNA for *Leptin* and *Serpine-1* (PAI-1 gene) was normalized to 18S rRNA. Values represent means ± SEM. Values with different superscripts are significantly different from each other, n = 6 per group. *p <*0.05, assessed by ANOVA.

To further characterize the role of RIP3 kinase in generating the adipose secretome induced by FFC diet, an adipokine array was used to assess the content of multiple adipokines in circulation. Of the 38 adipokines detected (**Figure 7A**), leptin and plasminogen activator inhibitor-1 (PAI-1) were increased the most in FFC diet-fed WT mice; this response was abrogated in *Rip3^K51A/K51A^* mice (**Figure 7B**). qPCR confirmed that *Rip3* kinase deficiency prevented FFC diet-induced expression of mRNA for *Leptin* and *Serpine 1*, the gene coding for PAI-1(**Figure 7C**). In previous studies, RIP3 can interact with PI3K/AKT signaling in mediating cellular events, including TNF-mediated cell death (27) and vascular smooth muscle cell growth (28). Moreover, TSC1/mTOR was found to control RIPK3-dependent necroptosis in intestinal inflammation and cancer (29). Therefore, we speculate that RIP3 kinase might be involved in the regulation of signaling, such as PI3K/AKT and mTOR pathways, required for leptin production and secretion from adipose tissue, both at baseline and in response to FFC diet feeding. Overall, deficiency of *Rip3* kinase attenuates FFC diet-induced adipose inflammation and accumulation of adipokines in the circulation.

## Discussion

The prevalence of obesity has steadily increased over the past few decades and has become a global health concern. Obesity is highly associated with the development of NAFL/NASH, with up to 70-80% of obese individuals having some degree of fatty liver disease (30). It is crucial to identify potential therapeutic targets in order to prevent disease progression at early stages of injury. In the current study, we found that both RIP1 and RIP3 kinases contribute to FFC diet-induced injury by affecting liver and adipose tissue functions in mice. Therefore, therapeutics designed to inhibit RIP1 and RIP3 kinase activity in hepatocytes would likely be beneficial in reducing liver injury associated with consumption of high fat diets and associated obesity.

The contribution of RIP1 and RIP3 to metabolic liver disease is complex and likely dependent on both domain-specific and cell-specific functional activity. RIP3 protein is composed of a C-terminal receptor-interacting protein homotypic interaction motif (RHIM) and an N-terminal kinase domain. The RHIM domain and nearby regions mediate the assembly of RIP3 oligomers, providing a scaffold for the RIP3 kinase domain to self-phosphorylate and/or phosphorylate its substrate MLKL (31). Emerging evidence indicates that RIP3 and MLKL play differential roles in different dietary models of NAFL/NASH, including the FFC model (8, 13). Current data indicated that *Rip3* kinase deficiency prevented FFC diet-induced liver injury. This is consistent with the protection of *Mlkl^-/-^* mice from FFC diet-induced liver injury, but not the lack of protection in *Rip3^-/-^* mice on this same diet. Taken together, these data suggest that other domains of RIP3 might counteract the effect of RIP3 kinase in response to high fat diets. Indeed, RHIM domain interactions with other RHIM domain-containing proteins, including RIP1, ZBP1 and TRIF, are a vital component of the signalling cascade and can also be involved in cell survival and death pathways in certain scenarios, particularly during viral and bacterial pathogen infection (32). The RHIM domain specific roles of RIP1 and RIP3 in NAFL/NASH need further investigation.

The necroptotic pathway is tightly regulated by multiple post-translational modifications, including phosphorylation and ubiquitination in different domains. These modifications play differential roles in cell survival and death pathways. For example, A20 inhibits K63-linked ubiquitination of RIP3 at K5 and disrupts the formation of necroptotic RIP1-RIP3 complexes (33). In addition, E3 ubiquitin ligases, including PELI1 and CHIP (targeting K363 in the intermediate domain) (34–36), and TRIM25 (targeting K501) (37), can induce K48-linked ubiquitylation of RIP3, leading to its proteasomal degradation. Apart from phosphorylation and ubiquitination, O-GlcNAc transferase (OGT)-mediated O-GlcNAcylation of RIP3 on T467 prevents RIP1/3 hetero- and RIP3/3 homo-interaction, thus dampening inflammation and necroptosis (38). Taken together these data demonstrate that different modifications in multiple domains of RIP3 can modify the role of RIP3 kinase in triggering necroptosis.

Given the distinct differences in the effect of *Rip3* deficiency and *Rip3* kinase inactive mutant in response to FFC feeding, we speculate that other kinases may become dominant and activate MLKL when RIP3 is completely absent. For example, in response to serum and amino acid deprivation, CAMK2/CaMKII, instead of RIP3, phosphorylates MLKL on the same sites (T357/S358 human; S345 murine) as RIP3 phosphorylation to facilitate autophagic flux (39). Members of the TAM (Tyro3, Axl, and Mer) family of receptor tyrosine kinases are also implicated in other phosphorylation sites of MLKL, include Y376 (human) and Y363 (murine) (40). In addition, recent studies reveal the role of MLKL ubiquitination in its necroptotic and non-necroptotic functions (41–43). Thus the emerging evidence indicates that the molecular mechanisms by which MLKL is activated in different conditions and cells in the presence/absence of RIP3 are complex and multifaceted.

In addition to domain-specific functions, it is also likely that cell-specific functions of RIP1-RIP3-MLKL play an important role in metabolic liver disease. For example, the RIP1-RIP3-MLKL-axis in immune cells plays a crucial role in the development of metabolic liver disease. A previous study demonstrated that RIP1 kinase activity in hematopoietic-derived macrophages contributes to hepatic inflammation and fibrosis progression in NASH (5). We also recently reported that macrophage-derived MLKL is essential for maintaining immune cell homeostasis and macrophage phagocytic function in alcohol-associated metabolic liver disease (44). While there was no difference in death of non-parenchymal cells in the liver, which include liver resident and infiltrating innate immune cells, between WT and *Rip3^K51A/K51A^* mice after FFC feeding, we cannot exclude the possibility that the RIP1 kinase-RIP3 kinase-MLKL-axis in hepatic immune cells plays a crucial role in the development of NAFL/NASH. Further studies utilizing cell-specific knockouts in the RIP1-RIP3-MLKL pathway would be beneficial to explore cell-specific roles of necroptotic cell death pathway in murine models of dietary-induced NAFL/NASH.

Domain-specific and cell-specific roles for RIP1-RIP3 may also be relevant to the impact of high fat diets and obesity in adipose tissue. RIP3 and pMLKL are induced in visceral white adipose tissue (WAT) in patients with obesity and are positively associated with body mass index and perturbed metabolic serum markers, such as HbA1c and insulin (14). However, the role of RIP3 in adipose tissue function is complex and poorly understood. *Rip3* deficiency does not affect white and beige adipocyte differentiation (45). RIP3 is thought to maintain adipose tissue homeostasis and dampen inflammation by inhibiting caspase-8-dependent apoptosis of adipocytes, associated with a prevention of glucose intolerance, in the choline deficient-HFD dietary model of obesity (14). However, bone marrow transplant studies revealed that immune cell-derived RIP3 in adipose tissue does not mediate the metabolic phenotype of choline deficient-HFD-fed model (14), suggesting a cell-specific role for RIP3 in non-myeloid cells, such as adipocytes, rather than immune cells, in adipose tissue. A domain-specific function of RIP3 is also indicated, since *Rip3^-/-^* mice are more susceptible to HFD and FFC diet-induced inflammatory responses in white adipose tissue (8, 12), but, in the current study, when only the kinase activity of RIP1 or RIP3 was eliminated, FFC diet-induced adipocyte death and adipose tissue inflammation were prevented (**Figure 6**).

In summary, *Rip1* and *Rip3* kinase deficiency prevented activation of MLKL and protected mice from FFC diet-induced liver injury and metabolic perturbations. Therefore, the RIP1 kinase-RIP3 kinase-MLKL-axis may serve as a potential target of small-molecule therapeutics by inhibiting pro-necroptotic pathway in the treatment of NAFL/NASH.

## Data availability statement

The original contributions presented in the study are included in the article/**Supplementary Material**. Further inquiries can be directed to the corresponding author.

## Ethics statement

The animal study was approved by Cleveland Clinic Institutional Animal Care and Use Committee. The study was conducted in accordance with the local legislation and institutional requirements.

## Author contributions

XW: Conceptualization, Data curation, Funding acquisition, Investigation, Methodology, Writing – original draft, Writing – review & editing. RA: Investigation, Methodology, Writing – review & editing. EH: Methodology, Writing – review & editing. MM: Project administration, Supervision, Writing – review & editing. LN: Conceptualization, Funding acquisition, Project administration, Resources, Supervision, Writing – review & editing.
