# Supplementary material for: Receptor-interacting protein 1 and 3 kinase activity are required for high-fat diet induced liver injury in mice
Source: Front Endocrinol (Lausanne). 2023 Dec 15;14:1267996. doi: 10.3389/fendo.2023.1267996 (PMC10757356; doi:10.3389/fendo.2023.1267996)
Supplement: Supplementary file 1 [file DataSheet_1.docx]

**Receptor-interacting protein 1 and 3 kinase activity are required for high-fat diet induced liver injury in mice**

Xiaoqin Wu^1^, Rakesh K. Arya^1^, Emily Huang^1^, Megan R. McMullen^1^ and Laura E. Nagy^1,2,3^

^1^Northern Ohio Alcohol Center, Department of Inflammation and Immunity, Cleveland Clinic, Cleveland OH

^2^Department of Gastroenterology and Hepatology, Cleveland Clinic, Cleveland, OH, United States;

^3^Department of Molecular Medicine, Case Western Reserve University, Cleveland, OH, United States

***Address correspondence to:***

Laura E Nagy

Cleveland Clinic

Lerner Research Institute/NE40

9500 Euclid Ave

Cleveland OH 44195

Phone 216-444-4021

Fax 216-444-9329

Email: [nagyL3@ccf.org](mailto:nagyL3@ccf.org)

**Supplementary data**

**Methods and materials**

**Animals and FFC diet feeding**

Male mice (5-6 weeks of age) were housed 2-3 per cage in standard microisolator cages and maintained in a temperature regulated facility with a 12h:12h light/dark cycle. Mice were allowed free access to a chow diet or FFC diet for 12 weeks. Mice were weighed weekly and food intake per cage measured weekly. Diet was changed weekly. At the end of the 12 week feeding protocol, mice were fasted for 4-6 hours prior to euthanasia. Portions of liver and epididymal adipose tissue were flash frozen in liquid nitrogen and stored at -80°C until RNA isolation or fixed in 10% formalin for histology. Blood was transferred to EDTA-containing tubes for the isolation of plasma. Plasma was then stored at -80°C. Lipid panel analysis, including blood glucose, insulin, cholesterol and triglyceride was performed by Marshfield Labs. HOMA-IR was calculated according to the formula: fasting insulin (microU/L) x fasting glucose (nmol/L)/22.5.

**Biochemical assays**

Plasma samples were assayed for alanine aminotransferase (ALT) and aspartate aminotransferase (AST) using commercially available enzymatic assay kits (Sekisui America Corp, Seacacus, NJ) following the manufacturer’s instructions. Total hepatic triglycerides were assayed using the Triglyceride Reagent Kit from Pointe Scientific Inc. (Lincoln Park, MI).

**Histopathology and immunohistochemistry**

Formalin-fixed tissues were paraffin-embedded, sectioned and stained with hematoxylin and eosin for histological analysis. Apoptosis was detected by using ApopTag Plus Peroxidase In Situ Apoptosis (S7101, EMD Millipore, Temecula, CA). Tissues were coded at the time of collection to assure an un-biased analysis; At least 3 images were acquired at 10X by cellSens (Olympus) per tissue section and 6 mice per experimental condition. Representative images are shown. Semi-quantification of positive staining was performed using cellSens and ImagePro Plus software (Media Cybernetics, Silver Springs, MD). No specific immunostaining for M30 was seen in sections incubated with PBS rather than the primary antibody (data not shown). Immunohistochemistry staining for pMLKL in paraffin-embedded liver sections. We have validated this specific IHC staining protocol using a negative control with *Mlkl*^-/-^ liver in previously published work [1].

**Isolation of RNA and quantitative real-time polymerase chain reaction (qRT-PCR)**

Total RNA was isolated from liver and adipose tissue and reverse transcribed followed by amplification using qRT-PCR. 10μl of reaction mix contained cDNA, Power SYBR Buffer (Applied Biosciences) and primers at final concentrations of 1 μM. qRT-PCR was performed in a Quantstudio 5 System for 40 cycles of 15 s at 95°C, 30 s at 60°C, 30 s at 72°C. The relative amount of target mRNA was determined using the comparative threshold (Ct) method by normalizing target mRNA Ct values to those of 18s. Primer sequences are listed in Supplemental **Table S1**.

**Adipokine array**

Plasma samples (n = 3/group) were pooled together and used for an Adipokine Array according to the manufacturer's instructions. (R&D Systems, Minneapolis, MN).

**MTS Cell Viability/Proliferation Assay**

Primary hepatocytes were isolated from male/female WT and *Rip3^K51A/K51A^* mice (12-16 weeks, WT mice were purchased from Jackson lab) by collagenase perfusion and suspended in William’s Media E with 10% FBS, pen/strep and primary hepatocyte maintenance supplement (Gibco, Grand Island NY). Isolated hepatocytes were plated in a 96 well plate and exposed to palmitic acid (PA) (P0500, Sigma) conjugated to bovine serum albumin (BSA) to model lipotoxicity. Reagents from CellTiter 96® AQueous One Solution Cell Proliferation Assay (G3581, Promega) was added to treated cells and placed in a 37°C incubator with 5% CO2 for 4 hour incuabation then record absorbace 490nm wavelength in a 96 well-plate reader.

**Localization of MLKL with phalloidin in primary hepatocytes**

Translocation of MLKL to the cell surface was assessed by confocal microscopy using phalloidin as a cell surface marker (phalloidin, A12380, Thermo Fisher) according to the manufacturer’s instructions. Cells were then washed twice with PBS and fixed with IC fix (Thermo Fisher) at room temperature for 10min. Coverslips were mounted with VECTASHIELD with DAPI reagent (H-1200, Vector Laboratories, Burlingame, CA) and fluorescent images were acquired using a 40× oil objective (zoom 4) by a Leica TCS-SP5II upright confocal/multi-photon microscope (Leica Microsystems, GmbH, Wetzlar, Germany). Co-localization of MLKL with phalloidin from 4 independent experiments (80-100 cells quantified) was analyzed by using Image Pro Plus v7.0.1. Values of the means ± SEM for co-localization area (yellow fluorescent area) per cell are presented with each image.

References

[1] X. Wu, K.L. Poulsen, C. Sanz-Garcia, E. Huang, M.R. McMullen, S. Roychowdhury, S. Dasarathy, and L.E. Nagy, MLKL-dependent signaling regulates autophagic flux in a murine model of non-alcohol-associated fatty liver and steatohepatitis. J Hepatol 73 (2020) 616-627.

**Table S1.** Primer sequences for qRT-PCR.

| Gene Name (Symbol) | Forward Sequence | Reverse Sequence |
| --- | --- | --- |
| *CXCL1* | TGCACCCAAACCGAAGTC | GTCAGAAGCCAGCGTTCACC |
| *Leptin* | CTCCATCTGCTGGCCTTCTC | CATCCAGGCTCTCTGGCTTCT |
| *PAI-1* | TTCAGCCCTTGCTTGCCTC | ACACTTTTACTCCGAAGTCGGT |
| *IL-10* | GGCGCTGTCATCGATTTCTC | TGCTCCACTGCCTTGCTCTTA |
| *Cd11c* | CTGGATAGCCTTTCTTCTGCTG | GCACACTGTGTCCGAACTCA |
| *C3aR* | TCGATGCTGACACCAATTCAA | TCCCAATAGACAAGTGAGACCAA |
| *C5aR* | GTGGGTTTTGTGTTGCCTCT | TGATAGGGCAGCCAGAAGAT |
| *Il-1β* | ATGGCAACTGTTCCTGAACTCAACT | CAGGACAGGTATAGATTCTTTCCTTT |
| *Il-6* | TAGTCCTTCCTACCCCAATTTCC | TTGGTCCTTAGCCACTCCTTC |
| *Mcp-1* | AGGTCCCTGTCATGCTTCTG | TCTGGACCCATTCCTTCTTG |
| *Tnfα* | CCCTCACACTCAGATCATCTTCT | GCTACGACGTGGGCTACAG |
| *F4/80* | CCCCAGTGTCCTTACAGAGTG | GTGCCCAGAGTGGATGTCT |
| *18S* | ACGGAAGGGCACCACCAGGA | CACCACCACCCACGGAATCG |
| *SAA* | CAGACTTTTCCGCACCTTGGG TTT | AGTGGGTGGTGCTGAAGTACGATT |
| *CRP-1* | ACACCAGGATGAAGCTACTCACCA | CCCTTGGAAAGCCTCGTGAACAAA |
| *Stab2* | GGATCAGTGGTGACGGAACT | GTCTTTGCTGGAAGGTGAGC |
| *Havcr2* | ACCCTGGCACTTATCATTGG | AATCCTGACTGCTCCTGCAT |
| *Abca1* | ATAGCAGGCTCCAACCCTGAC | GGTACTGAAGCATGTTTCGATGTT |
| *LIX(CXCL5)* | TCCTCAGTCATAGCCGCAAC | GCTTTCTTTTTGTCACTGCCCA |
| *Srebp-1c* | AACGTCACTTCCAGCTAGAC | CCACTAAGGTGCCTACAGAGC |
| *Pparγ* | TCGCTGATGCACTGCCTATG | GAGAGGTCCACAGAGCTGATT |
| *Fabp4* | AAGGTGAAGAGCATCATAACCC | TCACGCCTTTCATAACACATTCC |
| *Fas* | AGCGGCCATTTCCATTGCCC | CCATGCCCAGAGGGTGGTTG |
| *Mgat* | TGGTGCCAGTTTGGTTCCAG | TGCTCTGAGGTCGGGTTCA |

**
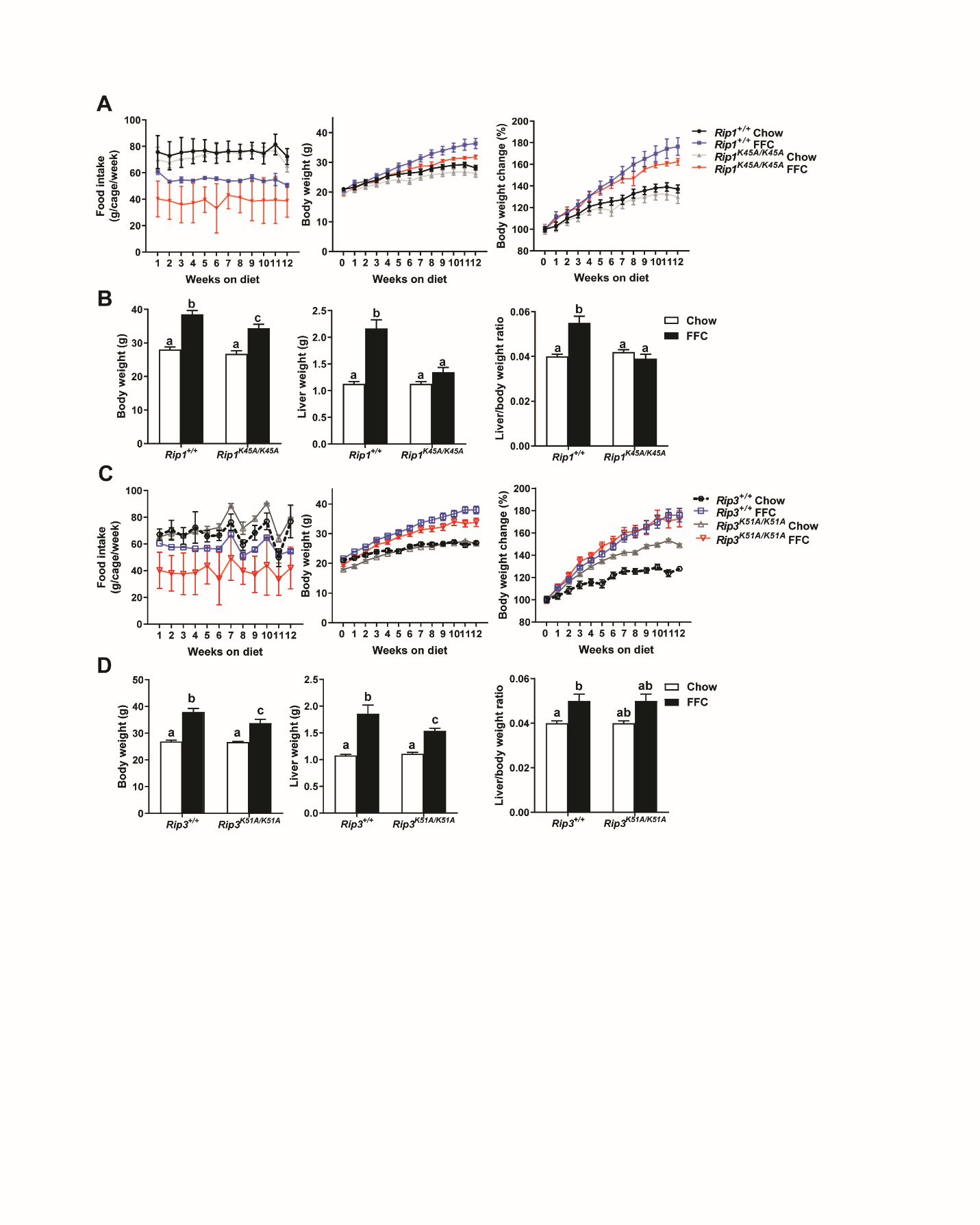
**

**Supplementary Figure S1. Food intake, body weight of different mouse strains on either chow or FFC diet.** *Rip1^K45A/K45A^* (n=8) and *Rip3^K51A/K51A^* (n=10) kinase-dead mice on a C57BL/6J background and their littermate controls (*Rip1^+/+^* or *Rip3^+/+^* n=8-12) were allowed free access to a diet high in fat, fructose and cholesterol (FFC diet) or chow diet for 12 weeks. (**A/C**) Food intake and body weight gain over 12 weeks. (**B/D**) Final body weight, liver weight and liver to body weight ratio.
